# Evaluation of Cytokeratin 19 Expression in Oral Carcinogenesis 

**DOI:** 10.30699/ijp.2025.2044451.3375

**Published:** 2025-07-01

**Authors:** Narges Ghazi, Nasrollah Saghravanian, Soheila Shamshiri, Yasamin Ayatollahi

**Affiliations:** 1 Department of Oral and Maxillofacial Pathology, School of Dentistry, Mashhad University of Medical Sciences, Mashhad, Iran; 2 Dentist, Mashhad, Iran

**Keywords:** Cytokeratin 19; Oral squamous cell carcinoma; Oral epithelial dysplasia; Immunohistochemistry; Biomarker; Oral carcinogenesis

## Abstract

**Background & Objective:**

Oral squamous cell carcinoma (OSCC) accounts for over 90% of oral cancers. Cytokeratin 19 (CK19), produced by suprabasal epithelial cells, reflects alterations in cellular behavior and has been associated with premalignant changes in the oral epithelium. This study aimed to evaluate the expression of CK19 in OSCC and oral epithelial dysplasia (OED) and its potential role in oral carcinogenesis.

**Methods:**

A total of 50 samples were analyzed, including 30 OSCC cases and 10 OED cases obtained from the archives of the Department of Pathology, as well as 10 normal oral mucosa samples collected from clinically healthy areas adjacent to mucocele lesions. CK19 expression was assessed using immunohistochemistry

**Results:**

Positive immunoreactivity for CK19 was observed in 90% (27/30) of OSCC samples. CK19 expression in the OSCC group was significantly higher compared to the OED and normal mucosa groups. Among OSCC cases, grade III tumors exhibited stronger CK19 expression than grades I and II. Additionally, CK19 expression in grade II OSCC was higher than in the OED group.

**Conclusion:**

The progressive increase in CK19 expression from normal mucosa to OED and OSCC supports its involvement in oral mucosal carcinogenesis. Moreover, higher CK19 expression correlated with increasing tumor grade and decreasing cellular differentiation, suggesting its potential value as a diagnostic and prognostic biomarker in OSCC.

## Introduction

Oral squamous cell carcinoma (OSCC) accounts for more than 90% of all oral malignancies and is histologically classified into grades I, II, and III. Among oral sites, tongue OSCC is associated with a higher frequency of lymphatic metastasis, nodal involvement, and poorer prognosis. Despite advancements in surgical techniques, radiotherapy, and chemotherapy, the 5-year survival rate for oral cancer patients remains low (1,2).

Oral epithelial dysplasia (OED) is histologically characterized by cellular and architectural alterations with malignant potential. Due to the risk of progression to carcinoma, accurate diagnosis of OED is a critical concern in oral pathology, underscoring the need for reliable histological and molecular markers. Numerous genes and proteins have been linked to oral carcinogenesis (4–6).

Cytokeratins, structural proteins of epithelial cells, are expressed specifically in epithelial tissues and their malignancies, such as OSCC (7). Cytokeratin 19 (CK19) was first identified by Wu et al. in 1981 as part of the cytoskeletal framework in cultured OSCC cells (8). Subsequently, Moll et al. (1982) characterized the expression profiles of various cytokeratins in both healthy and cancerous human tissues (9). Cytokeratins are water-insoluble proteins ranging in molecular weight from 40 to 70 kDa (10) and are divided into two groups: type I (acidic) and type II (neutral or basic) (11,12). CK19 is a low-molecular-weight, type I acidic cytokeratin (13) and has been used as a genetic and diagnostic marker in various malignancies, including breast (14), lung (15), and abdominal cancers (16,17). The CK19 gene, located on chromosome 17q21, consists of six introns and six exons and spans approximately 4.7 kb (8).

In the oral mucosa, the stratified epithelial architecture is maintained by continuous proliferation and differentiation of epithelial stem cells (ESCs) located in the basal layer. CK19, produced by these basal cells, serves as a marker for ESCs and reflects cell differentiation and maturation. In normal oral mucosa, CK19 expression is typically restricted to the basal layer and can be detected using immunohistochemistry. However, its expression in the suprabasal layers is considered indicative of altered cellular behavior and has been associated with premalignant transformation. In OSCC, CK19 expression may extend throughout the epithelial layers and is also seen in areas of carcinomatous invasion (7,17).

Nevertheless, there is inconsistency in the reported levels of CK19 expression in OSCC, with immunopositivity rates ranging from 29% to 100% (18–20). Furthermore, the relationship between CK19 expression and tumor grade or degree of pathological differentiation remains unclear.

Although several studies have explored CK19 expression in OSCC, few have assessed its progressive expression from normal mucosa through OED to various histological grades of OSCC. The present study aims to fill this gap by evaluating CK19 immunoreactivity across a spectrum of oral tissues, including normal mucosa, OED, and OSCC (grades I to III). This approach seeks to clarify CK19’s role in oral carcinogenesis and assess its potential as a diagnostic and grading biomarker. To the best of our knowledge, this is the first study in northeastern Iran to assess CK19 expression across both OED and histologically graded OSCC, highlighting its novelty and regional clinical relevance.

## Materials and Methods

### Sample preparation

A total of 50 formalin-fixed, paraffin-embedded tissue samples were analyzed in this study. These included 30 OSCC samples obtained from the Pathology Department archives, 10 normal oral mucosa samples taken from clinically healthy areas adjacent to mucocele lesions, and 10 cases of moderate oral epithelial dysplasia (OED) from patients clinically diagnosed with leukoplakia. Half of the OSCC samples were derived from the tongue, and the remaining half were from other intraoral regions. Each study group—normal mucosa, OED, OSCC grade I, OSCC grade II, and OSCC grade III—contained 10 samples.

Case selection was based on clear histopathological diagnosis and the availability of well-preserved archival paraffin blocks. OSCC grading was performed according to the World Health Organization (WHO) classification, which is based on the degree of cellular differentiation, nuclear pleomorphism, mitotic activity, and keratin production. The OSCC samples were equally distributed among well-differentiated (grade I), moderately differentiated (grade II), and poorly differentiated (grade III) tumors. All OED samples were histopathologically confirmed as moderate dysplasia according to WHO criteria, considering both cytological and architectural changes such as nuclear enlargement, hyperchromatism, increased nuclear-to-cytoplasmic ratio, and loss of epithelial polarity.

Histopathological evaluations were independently performed by an experienced oral pathologist and verified by a second pathologist. In cases of discrepancy, a consensus diagnosis was reached to minimize interobserver variability.

### Immunohistochemical staining

Sections of 4 µm thickness were cut from each paraffin block for immunohistochemical staining. The CK19 monoclonal antibody (NCL-L-CK19, Novacastra Laboratories, Newcastle, UK; dilution 1:50) was used following the manufacturer’s protocol. Staining was based on the antigen-antibody reaction. The immunoreactivity of epithelial cells was evaluated using a LABOMED microscope (USA) by examining 100 epithelial cells at 400× magnification in five representative high-density fields (hotspots).

Cells showing cytoplasmic staining were recorded and used to calculate the CK19 score.

### CK19 staining assessment

Staining intensity was categorized as follows: (–) no epithelial cells stained; (+1) 1%–25% of cells stained; (+2) 26%–50% of cells stained; and (+3) more than 50% of cells stained (17).

This study was conducted in accordance with the Declaration of Helsinki, and ethical approval was obtained from the Ethics Committee of Mashhad University of Medical Sciences. Informed consent was obtained from all participants prior to their inclusion in the study.

## Results

The study population had a mean age of 58.4 ± 15.4 years, with an age range of 20 to 90 years.

### Grade Distribution by Gender

The frequency of OSCC grades II and III was higher in women, while the frequency of grade I was similar between men and women. In contrast, oral epithelial dysplasia (OED) was more frequently observed in men. Despite these trends, the overall distribution of OSCC histopathological grades between genders did not show a statistically significant difference (p = 0.898).

**Table 1:** Distribution of lesion types by gender

				Groups				total	Fisher’s exact test
				OSCC I	OSCC II	OSCC III	OED		
Tongue	Gender	Male	Number	5	4	4	6	19	P=0.898
		Female	Number	5	6	6	4	21	
	Total		Number	10	10	10	10	40	

**Table 2:** Comparison of mean age across OED and different OSCC grades

	lesions	number	Mean age(years)	Standard deviation	Minimum(years)	Maximum(years)	one-way ANOVA test
total	OSCC I	10	57.3	16.0	32	77	F=0.03 P=0.991
	OSCC II	10	59.5	19.5	24	90	
	OSCC III	10	58.8	17.3	20	80	
	OED	10	58.0	9.6	42	76	

### Age distribution across groups

The lowest mean age was observed in grade I patients, while the highest was in grade II patients. However, no statistically significant difference in mean age was found among the four groups (OED, OSCC grades I, II, III) (p=0.991).

### Comparison of Immunostaining Patterns Among All Groups

Immunostaining intensity of epithelial cells was compared across five groups: normal oral mucosa, oral epithelial dysplasia (OED), and OSCC grades I, II, and III (n = 50). The results are summarized in Table 3.

All normal tissue samples exhibited low staining intensity (+1). In the OED group, most cases showed absent or weak staining, with 4 samples completely negative and 5 samples showing weak positivity. Only 1 case demonstrated moderate staining intensity (+2).

In contrast, CK19 immunostaining intensity increased progressively with the histopathological grade of OSCC. OSCC grade I samples showed a wide distribution across staining levels. OSCC grade II samples demonstrated a shift toward moderate and strong staining. OSCC grade III exhibited the highest intensity, with 8 out of 10 cases showing strong staining (+3) and none falling into the unstained or weakly stained categories.

A Kruskal–Wallis test revealed a statistically significant difference in staining intensity among the groups (χ² = 29.13, p < 0.001), indicating a meaningful association between CK19 immunoreactivity and histopathological progression.

In the pairwise comparison of groups, the mean staining score was significantly higher in the OSCC grade III compared to the OSCC grade I, OED and normal sample groups and also OSCC II compared to the OED group showed a higher score. No significant differences were found between the other groups (Table 4).

**Table 3:** The frequency of Immunostaining scores in all lesions and statistical results

			Immunostaining score				total
			unstained (0)	Up to 20% statining (+1)	20-50% staining (+2)	more than 50% staining (+3)	
Groups	OSCC I	number	3	2	4	1	10
	OSCC II	number	0	2	6	2	10
	OSCC III	number	0	0	2	8	10
	OED	number	4	5	1	0	10
	Normal	number	0	10	0	0	10
							50
Result of Kruskal-Wallis test			X2=29.13 p<0.001				

**Table 4:** Pairwise comparison of the groups in the study

Groups	Result
Normal and OED	P=1.00
Normal and OSCC I	P=1.00
Normal and OSCC II	P=0.148
Normal and OSCC III	P<0.001
OED and OSCC I	P=1.00
OED and OSCC II	P=0.026
OED and OSCC III	P<0.001
OSCC I and OSCC II	P=1.00
OSCC I and OSCC III	P=0.011
OSCC II and OSCC III	P=0.953

**Fig. 1:**
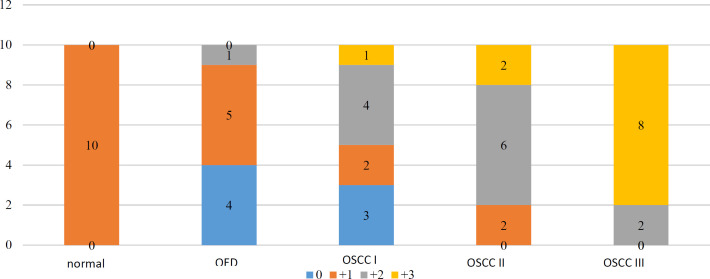
Distribution of CK19 scores across groups

**Fig 2:**
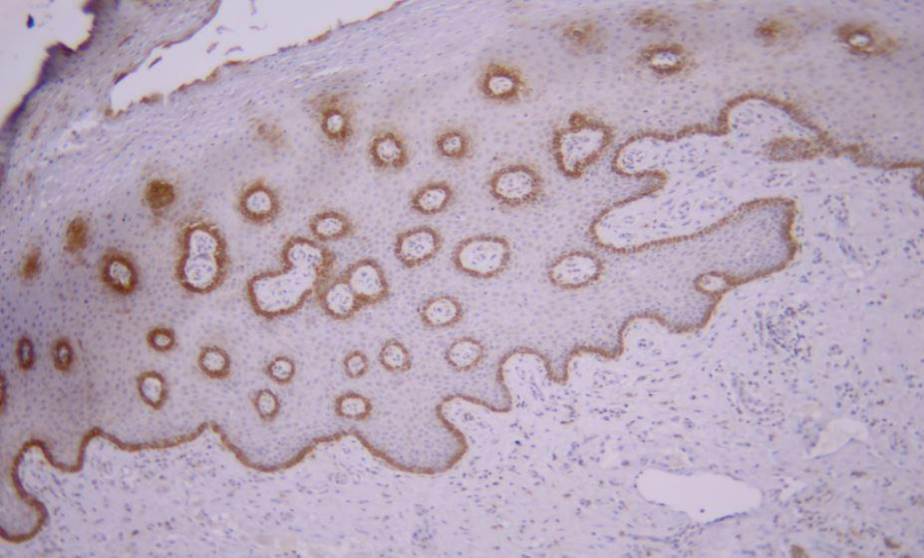
Expression of CK19 marker in the basal layer of normal mucosal epithelium (magnification 100X)

**Figure 3:**
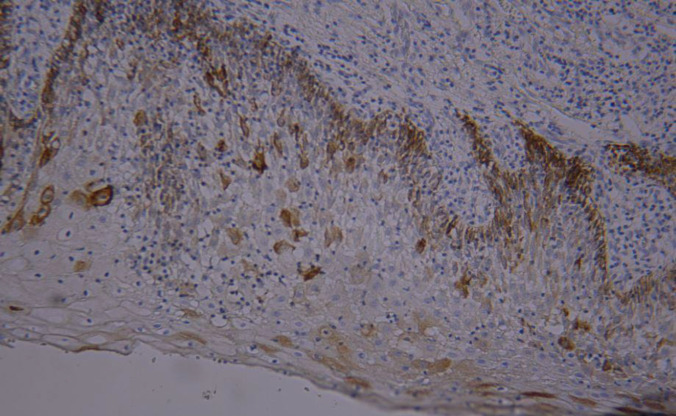
Expression of CK19 marker in OED (+1) (magnification 100X)

**Fig. 3:**
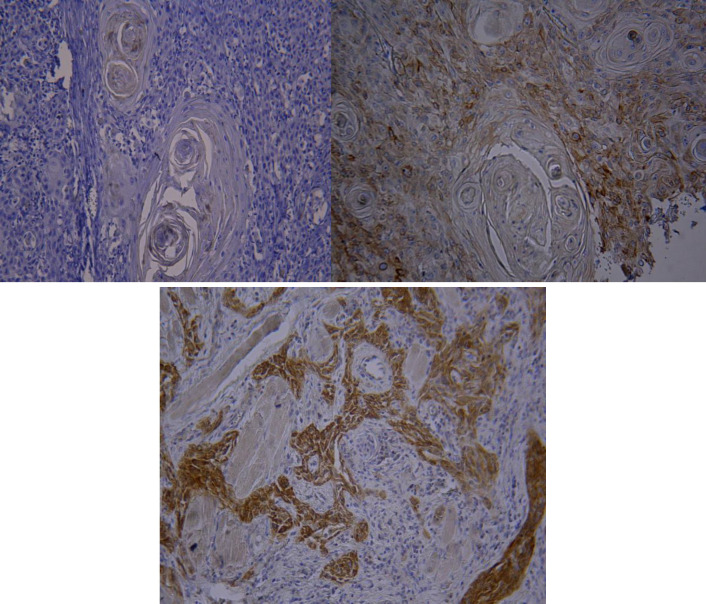
Expression of CK19 in A) OSCC grade I (+2) (magnification 100X), B) OSCC Grade II (+2) (magnification 400X), and C) OSCC Grade III (+3) (magnification 100X)

**Fig 4.**
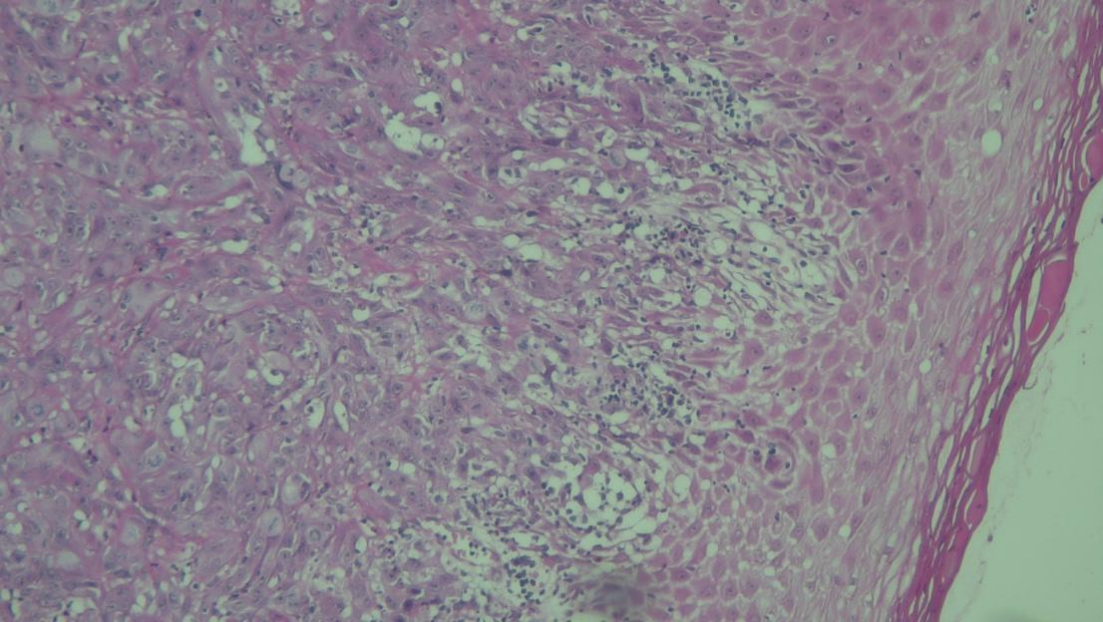
OSCC grade III stained with H&E (magnification 400X)

## Discussion

Cytokeratin 19 (CK19), a cytoskeletal protein, is specifically expressed in epithelial cells and their derived malignancies such as squamous cell carcinoma (SCC). While the role of CK19 has been widely studied, its clinical significance in the diagnosis and progression of oral squamous cell carcinoma (OSCC) remains an area of active investigation. Notably, increased CK19 expression in the suprabasal layers of the oral epithelium has been correlated with premalignant changes. In normal and hyperplastic oral mucosa, CK19 expression is typically confined to the basal layer, whereas in atypical hyperplasia, it extends into both basal and suprabasal layers (6–8, 18–21).

Lesions such as leukoplakia, which exhibit epithelial dysplasia, are recognized as potentially malignant oral lesions (PMOLs) and may undergo malignant transformation. Among the 21 known human cytokeratins—classified into type I and type II—mutations or altered expression patterns have been linked to various genetic disorders and pathological conditions (22). The layered structure of oral epithelium is maintained through the proliferation and differentiation of epithelial stem cells (ESCs) in the basal layer. CK19, produced by these basal ESCs, reflects the level of cellular differentiation and maturation. Its aberrant expression in SCC implies a possible disruption in ESC distribution and function, contributing to dysplastic transformation (23).

In the present study, CK19 expression varied significantly across normal mucosa, oral epithelial dysplasia (OED), and OSCC samples, with a clear increase in staining intensity associated with disease progression. Among OSCC cases, 90% (27 out of 30) demonstrated positive CK19 immunoreactivity. This finding is consistent with previous reports that describe a wide range of CK19 expression in OSCC.

For example, Vora et al. reported 29% positivity in tongue SCC (19), while Hamakawa et al. observed CK19 expression in 66.7% of OSCC samples (18). In contrast, Nie et al. found CK19 expression in 100% of SCC cases (24). Histopathological correlations reported by Lindberg and Rheinwald suggested that CK19-positive tumors are typically well-differentiated, whereas CK19-negative cases tend to be poorly or moderately differentiated (8). However, other studies, including those by Hamakawa and Vora, noted lower CK19 expression in highly differentiated carcinomas (18, 19, 24).

Rajeswari et al. (2021) examined CK19 expression in a range of samples, including different grades of OED, OSCC, and normal mucosa. Their results supported the hypothesis that increased CK19 expression may serve as a predictor of malignant transformation (25). Similarly, Yuchen Shen et al. (2022) reported CK19 positivity in 17 of 30 OSCC patients, with significantly higher expression compared to healthy controls (26).

In our immunohistochemical analysis, a statistically significant difference in CK19 expression was observed among OSCC grades, with grade III tumors showing three to five times greater staining intensity than grades I and II. Additionally, CK19 expression in OED was detectable but lower than in all OSCC groups. This pattern suggests an inverse relationship between the degree of differentiation and CK19 expression, with poorly differentiated tumors exhibiting higher CK19 levels. These findings support the potential role of CK19 as a histological and prognostic marker.

Beyond OSCC, CK19 has been recognized as a valuable biomarker in several other malignancies, including breast (14), lung (15), and gastric (16) cancers. In the context of OSCC, CK19 expression in peritumoral tissue has been associated with higher recurrence rates and reduced survival, further emphasizing its clinical relevance (5). Considering the function of CK19 as a putative stem cell marker and its apparent involvement in oral mucosal carcinogenesis, further large-scale studies are warranted to explore its mechanistic role and potential diagnostic applications.

## Conclusion

The progressive increase in cytokeratin 19 (CK19) expression from normal mucosa to oral epithelial dysplasia and through the various histological grades of OSCC highlights its potential role in oral carcinogenesis. A significant correlation between CK19 expression and disease progression was demonstrated, suggesting its involvement in both the development and advancement of OSCC.

Markers evaluated in this study showed high sensitivity, specificity, and diagnostic accuracy in distinguishing OSCC and OED from normal mucosa. Furthermore, CK19 expression effectively differentiated OSCC from OED, underscoring its potential as a valuable diagnostic tool for the early detection and histological classification of oral lesions.
